# Differential Cathelicidin Expression in Duodenal and Gastric Biopsies from Tanzanian and German Patients

**DOI:** 10.1371/journal.pone.0022049

**Published:** 2011-07-19

**Authors:** Dorothee Rogoll, Juergen Schauber, Koy K. Mheta, August Stich, Wolfgang Scheppach

**Affiliations:** 1 Department of Medicine II, University of Wuerzburg, Wuerzburg, Germany; 2 Department of Dermatology, University of Munich, Munich, Germany; 3 Bugando Medical Centre, Mwanza, Tanzania; 4 Department of Tropical Medicine, Medical Mission Hospital, Wuerzburg, Germany; 5 Juliusspital Wuerzburg, Wuerzburg, Germany; Charité, Campus Benjamin Franklin, Germany

## Abstract

**Background:**

Epithelial surfaces such as the gastrointestinal mucosa depend on expression of antimicrobial peptides like cathelicidin for immune defence against pathogens. The mechanisms behind mucosal cathelicidin regulation are incompletely understood.

**Methods:**

Cathelicidin expression was analysed in duodenal, antral and corpus/fundic mucosal biopsies from African and German patients. Additionally, cathelicidin expression was correlated with Helicobacter pylori (HP) infection and the inflammatory status of the mucosa.

**Results:**

High cathelicidin transcript abundance was detected in duodenal biopsies from African subjects. On the contrary, cathelicidin mRNA expression was either undetectable or very low in tissue specimens from German patients. Also, in the antrum and corpus/fundus regions of the stomach significantly higher cathelicidin transcript levels were measured in Tanzanian compared to German patients. In gastric biopsies from African patients cathelicidin expression was increased in HP positive compared to HP negative subjects. Additionally, the inflammatory status measured by IL-8 expression correlated well with the HP infection status.

**Conclusions:**

A higher duodenal and gastric cathelicidin expression in African (compared with European) individuals may be due to upregulation by antigenic stimulation and may confer a higher resistance against enteric infections.

## Introduction

The single cell layer of the colonic epithelium is an active barrier against the external environment and the enormous load of intestinal bacteria. In addition to forming a physical barrier, the epithelium is armed with an array of effector molecules including antimicrobial peptides [1]. These peptides can be considered as endogenous antibiotics and are widespread in nature as immediate defense effectors. They have been found in invertebrates, vertebrates, plants as well as bacteria, and several human antimicrobial peptides have been characterized [2].

The cathelicidins constitute a family of precursor proteins with a well conserved cathelin pro-region, followed by a highly variable C-terminal antimicrobial domain. The only human cathelicidin gives rise to LL-37, a 37-residue mature antimicrobial peptide, after cleavage from the cathelin propart. Both purified and chemically synthesized LL-37 peptides exhibit potent and comparable antimicrobial activities in vitro [3]. Antimicrobial peptides are active effector molecules in intestinal mucosal defense and constitute an integral part of immediate responses at epithelial barriers [4].

There are few papers on LL-37 expression in the upper gastrointestinal tract. In agreement with previous studies, increased expression of hCAP-18/LL-37 was observed in gastric mucosa obtained from Helicobacter pylori (HP) infected subjects [5], [6]. These authors concluded that cathelicidin contributes to determining the balance between host mucosal defence and HP survival mechanisms that govern chronic infection with this gastric pathogen.

It was hypothesized that constant contact with enteric pathogens in African patients might upregulate innate immunity of the host. Thus, LL-37 expression was assessed in mucosal biopsies from the duodenum, antrum, and corpus/fundus from Tanzanian patients and compared with those of German patients reporting for upper gastrointestinal endoscopy. Additionally, cathelicidin expression was correlated with Helicobacter pylori (HP) infection and the status of mucosal inflammation in African individuals.

## Results

**Figure 1:**
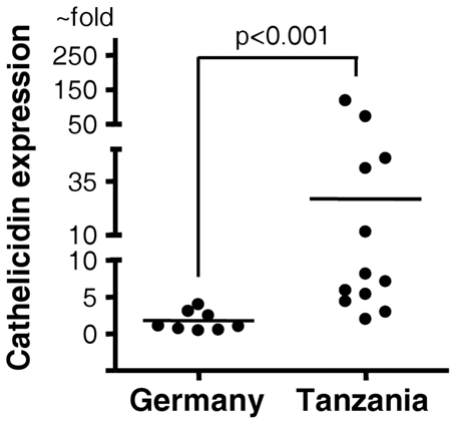
Expression of cathelicidin mRNA in human duodenal biopsies. Duodenal biopsies from 9 German and 12 African patients were analyzed by real-time PCR for cathelicidin mRNA expression. Levels are normalized to glyceraldehydes-3-phosphate dehydrogenase (GAPDH). Data are expressed as ∼fold change in mRNA transcript levels relative to German subjects. Horizontal bars represent median cathelicidin expression.

High cathelicidin transcript abundance was detected in the duodenal biopsies of African subjects. On the contrary, cathelicidin mRNA expression was either undetectable or very low in tissue specimens from German patients (Fig. 1). In addition, cathelicidin expression was also assessed in duodenal biopsies from five German travellers who had recently returned from Africa with diarrheal diseases (data not shown). Cathelicidin transcript levels were similarly undetectable or low as in German subjects without a history of overseas travel. Patient data are given in table 1.

**Table 1:** Clinical data of patients.

	Wuerzburg (Germany)	Mwanza (Tanzania)
Age	57,1+21,3 years (SD)	43,2+14,6 years (SD)
Sex	15 male, 20 female	18 male, 12 female
Endoscopic findings	hiatal herniareflux esophagitisBarrett's esophagusgastric ulcergastritisduodenal ulcerPEG placementno abnormality	hiatal herniareflux esophagitisesophageal varicesgastric ulcergastric cancergastritisduodenal ulcerduodenitisno abnormality
No. of biopsy sites:		
Duodenum	10	12
Antrum	10	27
Corpus and fundus	15	11

**Figure 2:**
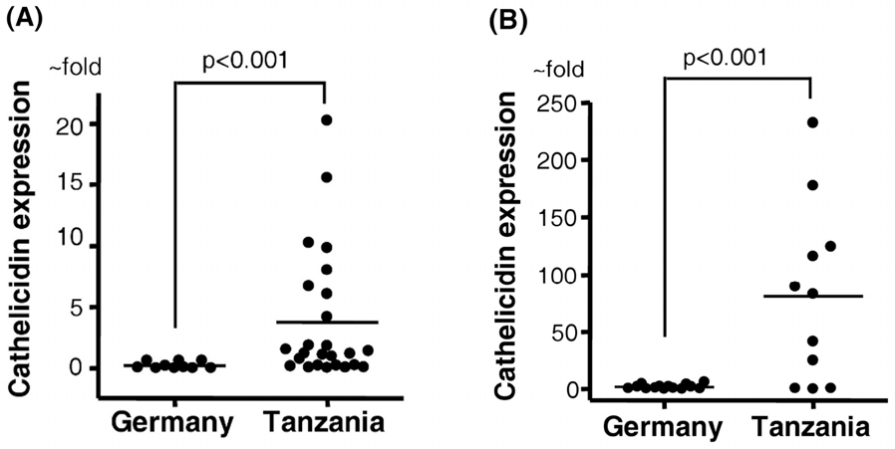
Expression of cathelicidin mRNA in biopsies from the stomach. (A) Biopsies from the antrum of 10 German and 27 African patients were analyzed by real-time PCR for cathelicidin mRNA expression. (B) Biopsies from the corpus and fundus of 15 German and 11 African patients were analyzed as described under (A). Levels are normalized to glyceraldehydes-3-phosphate dehydrogenase (GAPDH). Data are expressed as ∼fold change in mRNA transcript levels relative to German subjects. Horizontal bars represent median cathelicidin expression.

To determine the cathelicidin transcript level in the antrum and corpus/fundus regions we analyzed the gastric biopsies for LL37/hCAP18 mRNA expression. As shown in Figure 2A/B we measured significantly higher cathelicidin transcript levels in Tanzanian than in German patients (Fig. 2).

**Figure 3:**
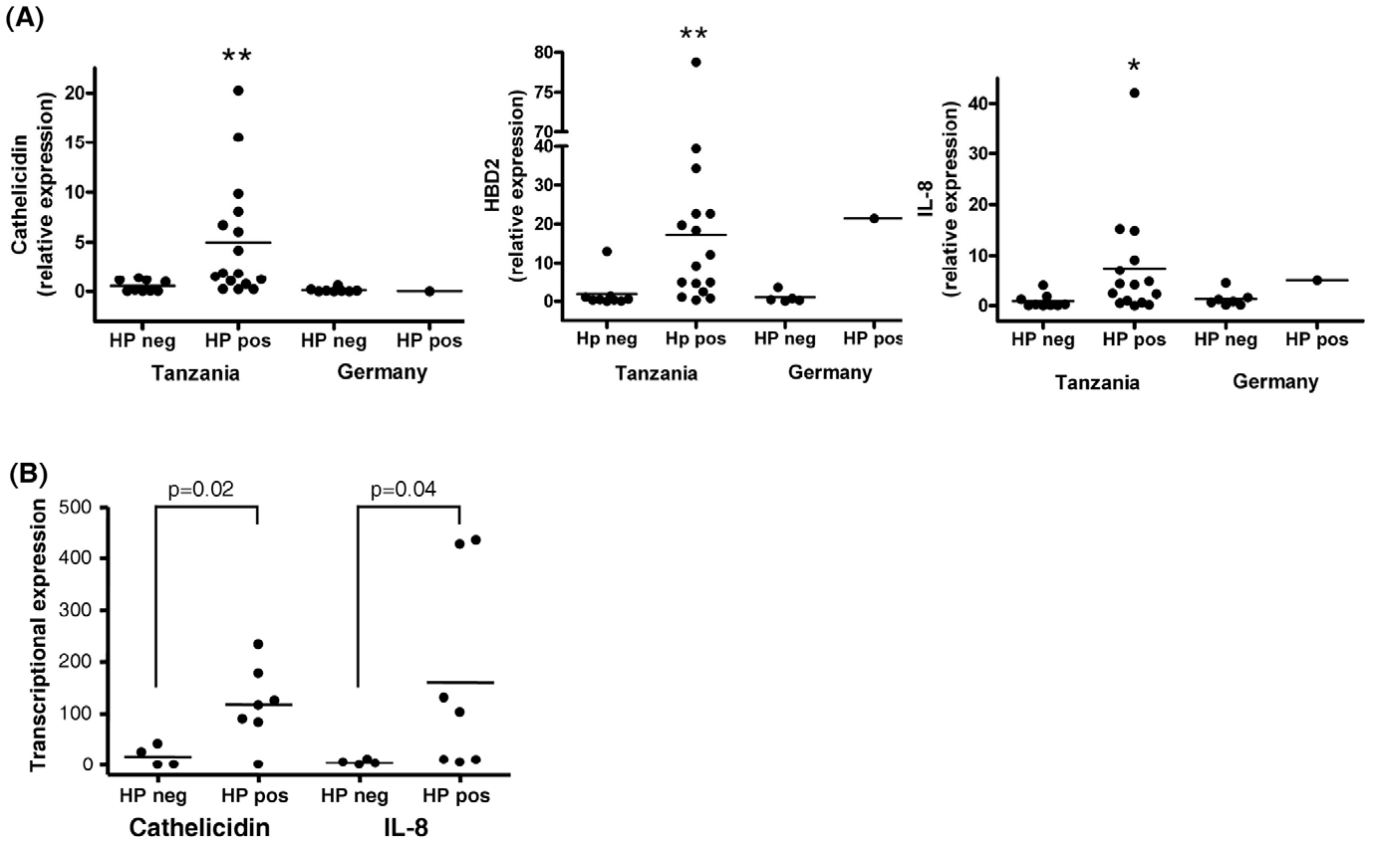
Expression of cathelicidin mRNA, interleukin-8 (IL-8) and beta-defensin 2 (hbd2) in biopsies. From the gastric antrum (A) and corpus/fundus (B) of African and German patients. Data were correlated with helicobacter pylori (HP) infection status. Levels are normalized to glyceraldehydes-3-phosphate dehydrogenase (GAPDH). Data are expressed as ∼fold change in mRNA transcript levels relative to German subjects. Horizontal bars represent median cathelicidin expression. Cathelicidin and hbd2 levels were significantly increased in HP positive compared with HP negative patients. The inflammatory status measured by IL-8 expression correlated well with the HP infection status.

In biopsies from African and German patients cathelicidin expression was analysed in relation to helicobacter pylori (HP) infection status: 19/27 african antral biopsies and 7/11 african corpus or fundus biopsies tested positive for HP. In contrast only 1/10 german antral biopsies and 3/20 corpus or fundus biopsies were tested positive for HP in Germany. As controls we also assessed mRNA levels from IL8 and hBD2, which was upregulated by mucosal inflammation. Figure 3A showed that cathelicidin, hBD2 and IL8 mRNA levels were significantly increased in HP positive compared with HP negative subjects. Figure 3B showed the african corpus/fundus biopsies correlated with the HP infections status. We could show that the IL8 as well as the hBD2 expression level correlated well with the HP infection status.

## Discussion

To protect the gastrointestinal tract, physical and chemical barriers (e.g. the production of mucin) or innate immune components of the blood (such as neutrophils, macrophages, etc.) are important. In addition to these diverse strategies, the active synthesis and secretion of small cationic peptides by the epithelium and immune cells has become recognized as a key mechanism for host defense. Antimicrobial peptides, e.g. defensins and cathelicidins, are small proteins that are considered together as a result of their antimicrobial activity but are in fact an extremely diverse group of gene products. Antimicrobial peptides participate in the innate immune system and are used by all eukaryotic organisms studied to date, including plants, insects and animals [7].

Cathelicidins are characterized by an N-terminal signal peptide, the cathelin prosequence, and a structurally variable cationic peptide at the C-terminus. The mature human cathelicidin is referred to as LL-37 because it begins with two leucine residues and is 37 amino acid residues long. Furthermore, the bactericidal activity of the human cathelicidin peptide requires proteolytic activation from its precursor. In-vitro studies using synthetic LL-37 show that this peptide has antimicrobial activity against Gram-negative and Gram-positive bacteria, with susceptible strains such as E. coli, P. aeruginosa, Klebsiella pneumoniae, Neisseria gonorrhoeae and group A Streptococcus. LL-37 and LL-37 derived peptides are also active against gastrointestinal pathogens, including several Helicobacter strains, Shigella, Salmonella and C. albicans [8].

Human cathelicidin is expressed by epithelial cells lining the stomach, lower small bowel and throughout the colon [9]. Expression is most prominent in the lower small intestine and colon with high peptide abundance in superficial epithelial cells. Nevertheless, except for butyrate, no microbial or inflammatory stimulus could be identified as being responsible for cathelicidin induction [10]. – High duodenal and gastric expression of cathelicidin in African individuals is a new finding as the bulk of available data has been obtained from subjects living in Europe or North America. The underlying mechanism is unknown; it may be speculated that constant exposure to enteric pathogens stimulates the mucosa of the upper gastrointestinal tract to upregulate the production of protective antimicrobial peptides and thus strengthen the barrier. – The expression of α-defensins has been measured in jejunal biopsies from Zambian adults; low mRNA levels were related to susceptibility to intestinal infection [11]. This paper is to our knowledge the only one in which data on innate immunity have been reported in an African cohort and it must kept in mind that this is a preliminary study that is applied to generate a hypothesis and confounding variables will be considered in future analysis.

Although the gastrointestinal tract is colonized by and exposed to a multitude of different bacteria, intestinal infection or translocation of bacterial agents is the exception, not the rule, and is mostly limited to highly pathogenic bacteria or predisposing disease states. Under normal conditions, the epithelial surface as well as the mucus layer of the colon are nearly devoid of bacteria, probably due to the constitutive epithelial synthesis of antimicrobial peptides. Evidence for a critical role for cathelicidin in antimicrobial defense at the gastrointestinal mucosa also comes from experimental animal models. Mice lacking cathelicidin are more susceptible to bacterial attachment to the intestinal wall [8]. Moreover, macrophages from cathelicidin deficient animals are unable to fight intracellular Salmonella bacteria [12].

In humans, intestinal cathelicidin expression is altered during infection, such as in the gastric mucosa during infection with H. pylori [5]. The finding of elevated cathelicidin expression in the presence of Helicobacter pylori is in line with the data of Hase et al. [5]. Human cathelicidin is scarcely expressed in the upper gastrointestinal tract of healthy Caucasians. These authors showed that surface gastric epithelial cells and epithelial cells in the fundic glands express cathelicidin peptide, and that the peptide is released into gastric secretions. Epithelial LL-37 expression is up-regulated by HP infection but not by proinflammatory cytokines. Furthermore, LL-37 has significant antimicrobial activity against several strains of HP. In agreement with recent studies our findings suggest that increasing production of bacterial peptide LL-37 may play a key role in host defence against HP. Leczczyńska et al conclude that increased expression of hCAP-18/LL-37 peptide in gastric mucus of infected subjects may have additional functions as an anti-inflammatory and growth stimulation agent. Alternatively, loss of defense against HP may be due to loss of antibacterial function of LL-37 in the milieu of gastric mucosa. In the antral biopsies, expression levels of hBD2 mRNA in the HP group were significantly increased in comparison to the non-HP group. LL37 and hBD2 have different bactericidal activity for HP. The various bioactivities of LL37 and hBD2 may depend on factors, e.g. differences in the membrane composition or structure of HP compared with *E. coli* [5].

There is scarce information on the regulation of antimicrobial peptides in the upper gastrointestinal tract. On the contrary, it is known that cathelicidin peptide is regulated physiologically in the colon by short-chain fatty acids such as butyrate [9]. Butyric acid is produced by the resident colonic flora and represents a major nutritional factor for colonocytes [13]. Consequently, in an attempt to fight Shigella infection, Raqib et al. [14] suggested that a disease such as shigellosis can be treated by stimulating the colon epithelium with butyrate to produce cathelicidin. In this landmark study, experimental dysentery was induced in rabbits and once symptoms were noted, the animals received butyrate or saline as a control. Significant benefit was observed from butyrate administration, including improvement in clinical symptoms, less blood in stool and reduction of bacterial counts in stools. These data indicate that cathelicidin is an essential antimicrobial factor in gastrointestinal epithelia and, for the first time, that therapeutic induction of cathelicidin expression might offer an alternative treatment option in the management of infectious disease of the intestine.

In order to survive in the human gastrointestinal tract, many bacterial pathogens have developed countermeasures to limit the effectiveness of antimicrobial peptides. Islam et al. [15] observed that Shigella can downregulate epithelial cathelicidin expression to subsequently invade the mucosa. This effect is mediated by plasmid DNA which is released from lysed Shigella dysenteria bacteria and penetrates the epithelial cell lining. A high basal expression level of antimicrobial peptides, as found in this study in Tanzanian individuals, may possibly be protective against invasive enteric organisms.

In conclusion, a better understanding of host defences will open up new therapeutic strategies to fight bacterial and other infections. The finding of higher duodenal and gastric cathelicidin expression in African (compared with European) individuals may explain a higher resistance against enteric infections and may stimulate research into new treatment modalities.

## Materials and Methods

### Patient data

Patients admitted to the University Hospital, Wuerzburg (Germany), and the Bugando Medical Centre, Mwanza (Tanzania), underwent routine upper gastrointestinal endoscopy with Olympus videoendoscopes (Wuerzburg) or fiberglass endoscopes (Mwanza). The endoscopic examinations were performed for various clinical reasons (tab.1); patients with diarrhea and suspected small bowel disease were not included in the study. At the University Hospital in Wuerzburg we included only German patients without ethnic African background (tourist or people from development aid programmes) in the study. Human studies were approved by an amendmend to the local ethics committee no. 104/00 (Medical Faculty, University of Wuerzburg, Germany), and all participants gave written consent prior to endoscopy. In Bugando/Tanzania informed consent was obtained for endoscopy including biopsies for scientific purpose.

Biopsies were taken with a standard forceps from the descending part of the duodenum, gastric antrum and corpus/fundus. They were placed immediately in RNA*later* medium (Ambion, Austin, Texas) and kept at room temperature until analysis within 4 weeks. Patient data are given in Tab. 1.

### RNA preparation and reverse transcription

Biopsies from 65 patients (30 African, 35 German) were disrupted in 1 ml RNA*later* medium (Ambion, Austin, Texas) until complete fragmentation as described previously [16]. Total RNA was extracted according to the manufacturer's RNA cleanup protocol (RNeasy, QIAGEN, Germany). Residual DNA was removed by on-column digestion during RNA purification with QIAGEN RNase-free DNase (QIAGEN). The RNA concentration was quantified by determination of the absorbance at 260 and 280 nm, and RNA integrity was checked by visualization on a 1.5% agarose gel. Subsequently, 1 µg RNA was reverse transcribed with iScript (BioRad, Hercules, California, USA).

### Real-time RT-PCR assays

For quantification the expression of cathelicidin and the housekeeping gene glyceraldehyde-3-phosphate dehydrogenase (GAPDH) were measured in triplicates by realtime RT-PCR using a BioRad iCycler (BioRad, Hercules, California, USA). Primers and fluorogenic probes for cathelicidin and GAPDH have been described previously [2]. Cathelicidin and GAPDH expression was measured by Ct and corresponding transcript numbers were read off standard curves as reported previously [9]. In addition, IL8 expression levels were quantified in all African and german patients using a Taqman real-time PCR assay (Applied Biosystems, Foster City, California, USA). Hbd2 expression levels were quantified using BDEFfor (5- GTATAATGTAAGGAAGGCGGGGAG-3) and BDEFrev (5- TCCTGGTTTCAACCT CATTCTTCT-3) from MWG (Germany) and BDEF probe (5–6FAM-ATGCTGCAAAAAG-MGBNFQ-3) from Applied Biosystems. Since in real-time PCR there is no established housekeeping gene for quantification of gene expression in biopsy material from different mucosa, relative levels of cathelicidin and IL8 transcription were calculated using two methods: gene transcript numbers were normalized to the amount of total RNA from Germany as recommended previously [9], [17], [18]; in addition, gene expression levels were normalized to GAPDH expression.

### Analysis of Helicobacter infection status

Total cDNA of the biopsies was used as template DNA to PCR-amplify the *H. pylori* 16 S rRNA gene. The PCR primers used are: 16S-5 GCTAAGAGATCAGCCTATGTCC and 16S-3 TGGCAATCAGCGTCAGGTAATG [19]. The resulting PCR product was 520 bp and showed a positive HP infection status of the patients.

### Statistical analysis

All statistical analyses were performed using SigmaStat 2.03 (SPSS Inc., San Rafael, California, USA). Student's paired t-test was used for analyses of data obtained from experimental studies, the Mann–Whitney rank sum test and the Kruskall–Wallis ANOVA were used for statistical comparison of grouped patient data. Differences were considered significant at p-values of 0.05 or less.
